# The Evolving Landscape of PD-1/PD-L1 Pathway in Head and Neck Cancer

**DOI:** 10.3389/fimmu.2020.01721

**Published:** 2020-09-18

**Authors:** Xin-wei Qiao, Jian Jiang, Xin Pang, Mei-chang Huang, Ya-jie Tang, Xin-hua Liang, Ya-ling Tang

**Affiliations:** ^1^State Key Laboratory of Oral Diseases, Department of Oral Pathology, National Clinical Research Center for Oral Diseases, West China Hospital of Stomatology, Sichuan University, Chengdu, China; ^2^Department of Head and Neck Surgery, Sichuan Cancer Center, School of Medicine, Sichuan Cancer Hospital & Institute, University of Electronic Science and Technology of China, Chengdu, China; ^3^State Key Laboratory of Microbial Technology, Shandong University, Qingdao, China

**Keywords:** PD-1, PD-L1, head and neck, prognostic, immunotherapy

## Abstract

Over the past 10 years, cancer immunotherapy has made significant progress in multiple cancer types and has been gradually been applied to clinical cancer care, in which the programmed cell death protein-1 (PD-1)/programmed cell death ligand 1 (PD-L1) pathway is one of the most attractive targets. Compared with traditional therapies, the emerging PD-1/PD-L1 blockade immunotherapy exhibited more satisfactory curative effects and lower toxicity for patients with advanced head and neck squamous cell carcinoma (HNSCC). This review analyzes the expression characteristics and clinical significance of PD-1/PD-L1 in HNSCC, the immunosuppressive roles of tumor cell and stromal cell expressing PD-1/PD-L1 in this disease, and presents the development landscape of PD-1/PD-L1 inhibitors, which may provide new curative alternatives for recurrent or metastatic HNSCC.

## Introduction

Head and neck cancers (HNC) are the sixth most common malignancies worldwide, with more than 680,000 new cases diagnosed every year (1, 2). Due to various carcinogenic factors, such as smoking, alcohol abuse, human papillomavirus (HPV) infection, and extended life expectancy, the incidence of HNC is rising year by year, of which the most frequent pathological type is squamous cell carcinoma (3). Although traditional therapies, including surgery, radiotherapy, and chemotherapy have made some progress in recent years, the prognosis for HNSCC patients remains unsatisfactory, with a 5-years survival rate of ~50% (4). More than 50% of HNSCC patients have had tumor recurrence and metastasis within 3 years (5). Therefore, it is necessary to optimize therapeutic regimes to improve the outcome of this disease. Over the past 10 years, cancer immunotherapy has made significant progress and has gradually been applied to clinical care in multiple cancer types. Various immunotherapeutic methods for HNSCC are under investigation, such as immune checkpoint inhibitors (ICIs), tumor vaccines, cell-based therapies, and cytokines therapy (6–8). At the same time, immunotherapy combined with other traditional therapies has also achieved improved curative effects for HNSCC patients in various clinical trials, which indicates that the clinical care of HNSCC is entering a new era (9–11).

Compared with the traditional treatments, the up-and-coming anti-PD-1/PD-L1 agents present better efficacy and lower toxicity for patients with advanced HNSCC (12–15). Based on the results of the clinical trial KEYNOTE-048, on June 10, 2019, the U.S. Food and Drug Administration (FDA) approved the PD-1 monoclonal antibody pembrolizumab (Keytruda) as the first-line therapeutic drug for patients with metastatic, unresectable, and recurrent HNSCC. Besides, FDA recommended Pembrolizumab in combination with platinum and fluorouracil for all advanced HNSCC patients, and as monotherapy for patients whose PD-L1 expression Combined Positive Score (CPS) is ≥1% (16). However, unlike Hodgkin's lymphoma in which the objective response rate (ORR) is as high as 87%, the ORRs of nivolumab and pembrolizumab are only 15% for HNSCC, indicating that much more effort should be made to investigate the pattern and mechanisms of PD-1/PD-L1 expression in HNSCC (17). And treatment-related adverse events have happened to over 50% of patients, which also impacted clinical outcomes (18). Herein, this article analyzes the expression characteristic and clinical significance of PD-1 and PD-L1 in HNSCC, focuses on how tumor cells and stromal cells expressing PD-1 and PD-L1 play immunosuppressive roles in HNSCC, and reviews the present development landscape of PD-1/PD-L1 inhibitors, which may be useful to HNSCC patients with recurrence and metastasis.

## Expression Features of PD-L1 and PD-1 in HNSCC

Belonging to the CD28 family, PD-1 (CD279) is one of the T-cell co-inhibitory receptors, expressing on various immune cells, such as activated T cells, regulatory T cells (Tregs, CD4^+^ Foxp3^+^), natural killer cells (NK cells), activated B cells and macrophages (19). PD-1 has two known ligands, PD-L1(B7-H1/CD274) and programmed cell death ligand 2 (PD-L2/CD273), from which PD-L1 is mainly expressed on T cells, B cells, dendritic cells (DCs), and macrophages (20, 21). Moreover, PD-L1 is also expressed in non-immune cells, such as cornea cells, vascular endothelial cells, mesenchymal stem cells, and keratinocytes, and it is often inducibly or constitutively upregulated on tumor cells of lots of solid and hematologic tumors (20). Interestingly, PD-L1 has been found in a soluble form (sPD-L1) in the patients' serum with tumors, such as melanoma and lung adenocarcinoma (22–25). Theodoraki et al. also demonstrated that sPD-L1 was found in plasma of patients with HNSCC (26). Besides, PD-L1 also binds to CD80 (B7-H1), which delivers inhibitory signals in T cells (27, 28). The expression of PD-L2 is more restricted to antigen-presenting cells (APCs), such as dendritic cells, macrophages, and B cells (29, 30). Similar to PD-L1, PD-L2 inhibits T-cell activation, decreases cytokine production, and induces T-cell cytolysis (31, 32). Nevertheless, in most cases, the PD-L2 expression was not detected in HNSCC tumor parenchyma according to Yearley et al. and Schoenfeld et al. (29, 33).

The over-expression of PD-L1 in tumor cells results from intrinsic and extrinsic regulatory mechanisms, in which IFN-γ secreted by immune cells is the most known potent cytokine inducer (34). A study found that human oral squamous cell carcinomas (OSCC) cell lines expressed various levels of PD-L1, and IFN-γ stimulation up-regulated PD-L1 expression on OSCC cells (35). Chen et al. found that IFN-γ induced PD-L1 expression by upregulating protein kinase D isoform 2 (PKD2), a downstream target of phosphoinositide 3-kinase (PI3K), in a time and dose dependent manner in OSCC cells (36). And inactivation of tumor suppressor gene phosphatase and tensin homolog (PTEN), which was often observed in human SCC, has been related to enhanced PD-L1 expression in lung SCC, representing the intrinsic mechanism of PD-L1 expression in tumors (37). Additionally, Chen et al. demonstrated that CMTM6, a type-3 transmembrane protein, induced PD-L1 expression in HNSCC cells and reduced CD8^+^ and CD4^+^ T cell infiltration (38). Others demonstrated that CMTM6 protected PD-L1 from ubiquitination in tumor cells and increased PD-L1 protein half-life (39, 40). At present, more attention has been paid to the intrinsic regulatory mechanism of PD-L1 expression on tumor cells.

Immunostaining of PD-1 shows high levels in inflammatory cells of HNSCC, especially at the invasive front of the tumor (41). In HNSCC tissues, PD-L1 showed both membrane and the cytoplasm staining by immunohistochemistry (IHC) (42). Besides, both tumor parenchyma and stroma showed PD-L1 expression, and increased PD-L1 expression in tumor parenchyma was associated with increased stromal expression (33). The expression of PD-L1 has two patterns, diffuse staining throughout tumor parenchyma from an overview of the tumor, or peripheral staining around tumor parenchyma. The majority was peripheral staining on both tumor cells and tumor-associated macrophages (TAM), especially in front of tumor parenchyma, which may be related to the inflammatory microenvironment and invasion front of HNSCC (43, 44). The degree of PD-L1 expression in HNSCC tissues is varied from study to study, which may be for several reasons, such as different protocols for immunohistochemical staining, different antibodies with varying binding affinities, the inconsistent cut-off for positivity, biopsy quality, subjective factors of pathologists' evaluation and intratumor heterogeneity (45). These studies are shown in Table 1.

**Table 1:** PD-L1 positivity in HNSCC of previous studies.

**Study**	**Sample size**	**Cut-off of PD-L1 positivity**	**% Positive**	**% Positive in HPV^**+**^ vs. HPV^**−**^**	**Tumor types**
Lyford-Pike et al. (44)	27	5% of tumor cells	59%	70 vs. 29%	Oropharyngeal SCC
Kim et al. (46)	133	20% membrane staining of tumor cells	68%	61 vs. 71% (*p* = 0.274)	Oropharyngeal SCC
Hong et al. (49)	99	Any unequivocal membrane staining of tumor cells	69.7%	83.3 vs. 56.9%	Tonsillar cancer
Mattox et al. (43)	53	1% membrane staining of tumor and/or immune cells	79%	Not reported	Tougue SCC
Balermpas et al. (48)	161	5% of tumor and/or stromal cells	39.1%	53 vs. 31%	HNSCC (including oral cavity, oropharynx, and hypopharynx)
Ou et al. (47)	38	1% on both tumor and immune cells 5% on both tumor and immune cells	71.1% 50%	No significant relationship	HNSCC (multiple sites)

With HPV infection becoming a cause of a subset of HNSCC, of which the incidence is increasing year by year, many studies intent on finding out how the immune microenvironment of HPV-positive tumors is different from HPV-negative tumors, including the expression differences of PD-L1. Although few studies found no correlation between PD-L1 and HPV positivity (46, 47), most have indicated that PD-L1 expression levels are positively related to HPV infection (33, 44, 48–50). Moreover, a meta-analysis showed that PD-L1 positive expression accounted for 42% of 3,105 HNSCC patients and was associated with HPV status (51). These results suggest that PD-1/PD-L1 pathway plays a specific role in the pathogenesis and development of HPV-positive HNSCC.

## Biological Significance and Prognosis Value of PD-1/PD-L1 Axis in HNSCC

Since the PD-1/PD-L1 pathway is involved in immune evasion and tumor progression, many researchers have conducted in-depth research into whether the expression level of PD-1 and PD-L1 protein in tumor tissues is related to the clinical characteristics and biological behavior of HNSCC. However, the current results are still controversial. Some researchers concluded that stronger PD-L1 immunostaining in HNSCC tissues correlates with distant metastases and worse outcomes, independent of tumor origin (52). Similarly, Moratin et al. found that higher PD-L1 expression in OSCC was associated significantly with tumor size, clinical stage, regional metastases, as well as worse overall survival (OS) (53). Additionally, levels of PD-L1 that carried by circulating exosomes were positively correlated with the UICC stage and the lymph node status of HNSCC, indicating that PD-L1 expression in circulating exosomes may also be a metric for HNSCC (26).

On the contrary, others reported that higher PD-1/PD-L1 expression predicted a better outcome, with significantly fewer local and distant recurrences which was particularly prominent in HPV-positive patients (48, 54). In a study of tonsillar cancer, patients with both HPV and PD-L1 positivity had longer progression-free survival (PFS), OS, and lower risk of death (49). It was reported that HPV-positive HNSCC patients had high PD-1 expression, and the PD-1 high group in these patients who treated with radiotherapy had better recurrence-free survival (55). And the therapeutic response to immunotherapy was better in HNSCC patients with higher PD-L1 expression (56). Thus, the better outcome of PD-1-PD-L1^+^ HNSCC patients may be a result of better response to radiotherapy and immunotherapy.

However, Kim et al. reported that PD-L1 expression of tumor cells is not related to the clinical characteristics and prognosis of HNSCC patients (46). HNSCC patients whose tumor cells do not express PD-L1 still respond to treatment, which demonstrates that PD-L1 or PD-1 expression on non-tumor cells plays a specific role. A meta-analysis of PD-L1 expression detected by IHC in predicting survival of HNSCC patients suggested no significant difference in OS between PD-L1-positive and -negative HNSCC patients (51). While for patients with low CD8^+^ tumor-infiltrating T cells, a poorer OS was detected in those with positive PD-L1 expression than those with negative PD-L1 expression, showing that PD-L1 expression on immune cells rather than tumor cells was associated with a better outcome for HNSCC (51, 57). In a study of nasopharyngeal cancer, PD-1 expression was higher in CD8^+^ TILs than that in healthy tissues and correlated with poor prognosis (58). These indicated that PD-L1 or PD-1 expression on non-tumor cells may be useful for guiding treatment of HNSCC and the prognostic role of PD-L1 expression combined with immune cells infiltrating should be further investigated.

HPV infection can affect the host immune response and immune activation in HNSCC. Recent research has found that HPV-positive HNSCC showed a higher level of PD-1 mRNA and increased PD-1^+^ T cells, while the latter was associated with worse outcomes (59–61). However, Poropatich et al. found that a higher proportion of PD-1^+^ CD8^+^ T cells and cytotoxic T lymphocyte antigen 4 (CTLA-4)^+^ CD8^+^ T cells were present in the tumor tissues and peripheral blood of HPV-negative HNSCC, and PD-1^+^ CD8^+^ T cells were related to primary tumor size (62). Sufficient PD-1^+^ T cells subset represented the previous activation state of T cells against tumors, which could be reactivated by PD-1/PD-L1 blockade (57). Additionally, a histoepigenetic analysis showed that HPV-positive HNSCC had a higher level of both infiltrated CD8^+^ T and B cells in the tumors as well as higher PD-1 expression in immune cells, which may lead to a better response rate by PD-1 targeted therapy (63).

These inconsistent conclusions give cause for consideration. Some researchers pointed out that the extent of PD-1 expression rather than the frequency determined T-cell function and affected clinical outcome and response to PD-1/PD-L1 inhibitors. CD8^+^ TILs in HPV-negative HNSCC were mostly characterized by high-density of PD-1 expression, which symbolized a state of dysfunction accompanied by suppressed IFN-γ secretion, associating with worse disease-free survival (DFS) and higher hazard ratio for recurrence. In contrast, low-density PD-1 was predominantly expressed in T cells of HPV-positive HNSCC patients, who had a better outcome (64). Overall, recent research has been mainly focused on PD-1 or PD-L1 expression in tumor cells and T cells of HNSCC, but little is known in NK cells, macrophages, B cells, DCs, or other stromal cells.

## Mechanisms of the PD-1/PD-L1 Pathway in Tumor Evasion of HNSCC

**Figure 1:**
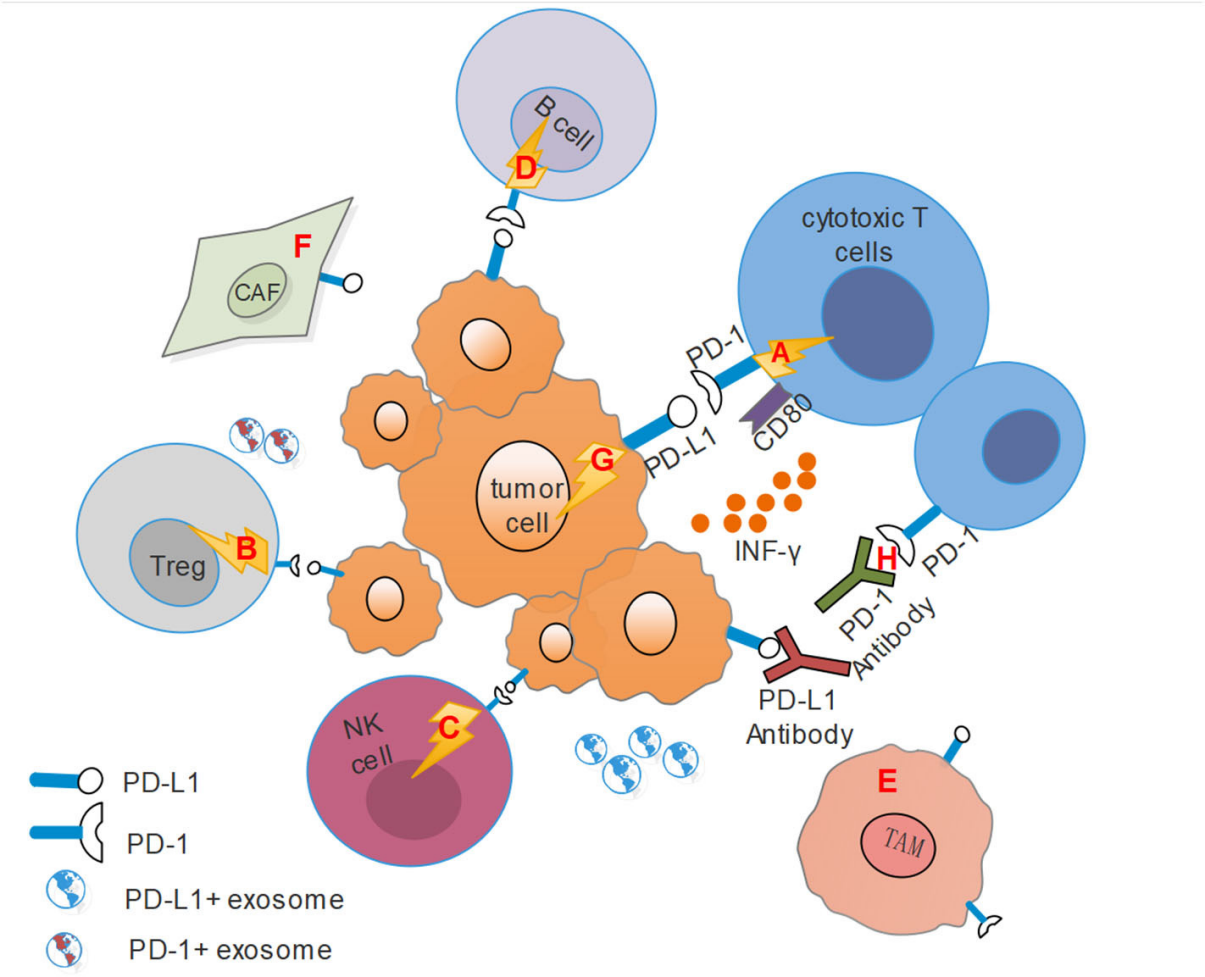
This figure shows the immunosuppressive microenvironment mediated by PD-1/PD-L1 pathway. CAF, Cancer-associated fibroblasts; TAM, Tumor-associated macrophages; Treg, regulatory T cells. **(A)** PD-L1 on tumor cells combines with PD-1 on cytotoxic T cells transmits an inhibitory second signal to T cells, causing effector T cells exhaustion, dysfunction and tumor progression. At the same time, PD-L1 can act as a ligand to bind with CD80 on effector T cells, competitively inhibits the binding of costimulatory molecule CD28 with CD80, and hinders T cell activation. **(B)** PD-L1 also binds to PD-1 on Tregs, resulting in immune suppression by raising the threshold for T-cell activation. **(C)** For NK cells, PD-1 represents an “activated” phenotype and binds to PD-L1 on tumor cells or stromal cells, leading to its dysfunction. **(D)** Activation of PD-1 signals on B cells can inhibit the proliferation of CD4^+^ and CD8 T^+^ cells. **(E)** Activated CD4^+^ T helper cells modulated the up-regulation of PD-L1 expression on macrophages via IFN-γ, and TAMs could mediate adaptive resistance and dampen tumor specific T cell function based on PD-L1 expression. **(F)** HNSCC tumor cells recruit fibroblasts and up-regulate PD-L1 expression on fibroblasts. Conversely, fibroblasts can increase PD-L1 expression on HNSCC cells. **(G)** PD-L1 on tumor cells binding with PD-1 can transmit anti-apoptotic signals to the tumor themselves. **(H)** PD-1 antibodies can competitively inhibit the binding of PD-1 to PD-L1, while PD-L1 antibodies bind to PD-1, and they both inhibit the activation of the PD-1/PD-L1 signal pathway and reverse the suppressive effect.

Studies have suggested that immune cell dysfunction within the tumor microenvironment with HNSCC resulted in immunosuppression (8, 65). PD-1/PD-L1 axis participates in modulating immune cells and other stromal cells and has been proposed as a potential mechanism for the formation of this immunosuppressive microenvironment, further facilitating immune evasion and immune resistance (Figure 1).

### T Cells

Inhibitory immune-checkpoint receptors (ICRs), such as PD-1, CTLA-4, and T cell Ig and mucin domain-3 protein (Tim-3), are considered to represent an exhausted and inactivated T cell phenotype in HNSCC (66, 67). Not only that, but PD-1 is also expressed in varying degrees on draining lymph nodes, circulating CD4^+^ and CD8^+^ T cells (68). Expression levels of various immune regulatory molecules, such as OX40, PD-1, PD-L1, and CTLA-4, are always higher in TILs than those in peripheral blood lymphocytes (PBL) (44, 69, 70).

APCs present antigens to T cells through the T cell receptor (TCR) and activate T cells. Binding of PD-L1 with PD-1 on activated T cells blocks PI3K and Akt activity, disrupts glucose metabolism and suppresses Th1 cytokines production, which could induce effector T cells dysfunction, exhaustion and protect tumor cells from being killed by CD8^+^ T cells (cytotoxic T cells) (71, 72).

Recent studies show that intratumoral Tregs suppress anti-tumor immunity and is involved in tumor progression. Compared with peripheral Tregs, inhibitory receptors PD-1, CTLA-4, and Tim-3 were significantly increased in tumor-infiltrated Tregs in HNSCC, signifying more suppressive function (59, 70). The interaction between PD-L1 and PD-1 on Tregs enhances the proliferation of Tregs and promotes the development of Tregs, leading to immune suppression by raising the threshold for T-cell activation. In the presence of transforming growth factor-β (TGF-β), PD-L1 can promote the conversion of naive CD4^+^ T cells to Tregs simultaneously improve the suppressive capabilities of the latter (73, 74). And recent research indicated that PD-1 expression on Tregs represents a state of “exhaustion,” and such Tregs were less suppressive. Tim-3 is also a regulatory molecule on intratumoral Tregs and represents the activation state of Tregs, which inhibits T cell proliferation. Furthermore, PD-1/PD-L1 blockade down-regulated the expression of Tim-3 on Tregs isolated from murine HNSCC tumors, which may be related to the release of IFN-γ (75).

### NK Cells

NK cells are of great importance in connecting innate and adaptive immunity, and also play a vital role in anti-tumor immunity. It was reported that HNSCC patients have high levels of PD-1^+^ NK cells within the tumor and peripheral blood. PD-1 expression on NK cells represents the activation status, and cetuximab-mediated NK cell activation can up-regulate PD-1 expression. However, once bound to its ligand PD-L1, such activation state is inhibited. In turn, PD-1 blockade can enhance cetuximab-mediated antibody-dependent cytotoxicity (ADCC), thus resulting in HNSCC cell lysis (76). Makowska et al. found that PD-1 blockade increased cytotoxicity of IFNβ-activated NK cells toward nasopharyngeal carcinoma cells, which resulted from the secretion of tumor necrosis factor-related apoptosis inducing ligand (TRAIL) (77).

### TAMs

TAMs are critical infiltrated inflammatory cells for cancer promoting inflammation (78). PD-L1 expression on macrophages delivers a constitutive negative signal, resulting in an immune-suppressive cell phenotype and contributing to the immune-suppressive tumor microenvironment (79). In a study on oral tongue squamous cell carcinoma (OTSCC), activated CD4^+^ T helper cells up-regulated PD-L1 expression on macrophages via IFN-γ (43). PD-L1 expression may provide a means to protect macrophages or DCs from cell death in OSCC (80). Besides, OSCC tumor cells induced PD-L1 expression on TAMs via IL-10 and led to T cell apoptosis and unfavorable prognosis (81, 82). Also, in HPV-positive HNSCC, TAMs could mediate adaptive resistance and dampen tumor-specific T cell function based on PD-L1 expression at the interface between the tumor nests and the surrounding inflammatory stroma (44). In turn, PD-L1 expression on tumor cells can be induced by macrophages in HPV-positive HNSCC (83).

### Cancer-Associated Fibroblasts (CAFs)

As part of tumor stromal cells, CAFs have received much attention in recent years and correlate with tumor invasion and metastasis. They secrete various growth factors, chemokines and proteases to regulate and recruit innate and adaptive immune cells. PD-L1 is also expressed on CAFs, and it has been reported that 40% of HNSCC tissues showed PD-L1 positivity on CAFs but showed no clinical significance (84). Baruah et al. found that HPV-positive HNSCC tumor cells recruited fibroblasts and up-regulated PD-L1 and PD-L2 expression on fibroblasts via the TLR9-mediated mechanism. Conversely, fibroblasts can increase PD-L1 expression on HPV-positive HNSCC cells (83). Nazareth et al. demonstrated that PD-L1 expressing human non-small cell lung cancer associated fibroblasts could inhibit the activation of T cells, and it could be completely abrogated by PD-L1 blockade (85). And research also suggested that TGF-β could induced human and murine lung fibroblasts PD-L1 expression, and further inhibited T cell proliferation in response to T cell receptor stimulation through secreting exosomes that contained PD-L1 (86). It is known that PD-L1 overexpression could induce T-cell apoptosis in tumor (87). Therefore, there is a mutual interaction between tumor cells and stromal cells, which finally increases the overall PD-L1 level, creating an immune-suppressive microenvironment. However, at present, whether PD-L1^+^ CAFs modulate T cell activity through direct contact or secreting cytokines is still unclear. Thus, the underlying mechanisms of PD-L1 induced or expressed by CAFs participating in remodeling the inflammatory microenvironment in HNSCC still need further investigation.

### Tumor Cells

A population of CD44^+^ tumor cells exists in HNSCC, which are considered “cancer stem cells” and are associated with tumorigenesis and resistance to chemo- or radiotherapy (88). Lee et al. found that CD44^+^ tumor cells preferentially expressed PD-L1 on CD44^+^ tumor cells rather than CD44^−^ cells, and was less immunogenic when cocultured with expanded autologous CD8^+^ TILs. While the impeded immunogenicity of CD44^+^ cells was reversed by PD-1 blockade. These suggested CD44^+^ tumor initiating cells could evade immune surveillance by expressing PD-L1 (89).

### Exosomes

Exosomes are 50–150 nm endocytic vesicles, carrying specific bioactive molecules that are secreted by multiple kinds of cells (90). Such small vesicles derived from tumor cells, which are called tumor-derived exosomes (TEX), are believed to be a new way for tumor cells to communicate with stromal cells (91, 92). Recently, different cell-derived exosomes have been isolated in HNSCC, including TEX and T cell-derived exosomes, and are considered to be associated with immune suppression in cancer (93). Compared with healthy people, HNSCC patients have higher levels of various exosomes that carry inhibitory proteins in plasma, such as PD-L1, CTLA-4, and cyclooxygenase-2 (COX-2), which can mediate immune suppression (94). Besides, PD-L1-positive exosomes were related to the disease activity, UICC grade, and lymph node levels of HNSCC patients. In contrast, sPD-L1 in plasma and PD-1-positive exosomes had no relation with clinicopathological features. Furthermore, exosomes carrying high levels of PD-L1 suppressed CD8^+^T cell activity. Thus, PD-L1^+^ exosomes can act as markers of tumor progression (26). The functional differences between exosomes derived from different cells and the underlying mechanism are still worthy of further study.

## The Clinical Application of PD-1/PD-L1 Inhibitors in HNSCC

There have been two PD-1 inhibitors approved by the FDA to treat advanced HNSCC so far—Nivolumab and Pembrolizumab. Besides, several clinical trials related to PD-L1 inhibitors—Durvalumab and Atezolizumab, have been completed or are still ongoing. To date, PD-1/PD-L1 blockade has been shown to reduce tumor growth and achieve durable tumor regression in HNSCC.

### Nivolumab

Nivolumab is a high affinity, fully human, IgG4 monoclonal antibody that inhibits the binding of PD-L1 to both PD-1 and CD80. In phase III clinical trial CheckMate141, Nivolumab showed a benefit on OS and lower toxicity compared to the standard treatment. Thus, in 2016, the FDA approved Nivolumab for the treatment of recurrent/metastatic HNSCC with or without PD-L1 expression. In this trial, compared with standard therapy (methotrexate, docetaxel, or cetuximab), Nivolumab prolonged median overall survival(mOS) of patients (7.5 vs. 5.1 months), but did not affect median progression-free survival (mPFS). The ORR in the Nivolumab group and standard therapy was 13.3 and 5.8%. The incidence of grade 3-4 adverse events (AEs) was 13.1 and 35.1%, respectively. Also, it was preliminarily observed that PD-L1-positive (cut-off ≥1%) and p16-positive tumors are more likely to benefit from nivolumab monotherapy (95).

### Pembrolizumab

In the phase I trial KEYNOTE-012, 60 HNSCC patients whose PD-L1 expression was positive were enrolled and then treated with Pembrolizumab monotherapy. 38% (23 of 60) of patients were HPV-positive, and 62% (37 of 60) were HPV-negative. The ORR was 18% (8 of 45 patients) in all patients, 25% (4 of 16 patients) in HPV-positive patients, and 14% (4 of 29 patients) in HPV-negative patients, which showed that HPV-positive HNSCC had a better curative effect. Besides, the mOS was 13.0 months, and the duration of response was ~53 weeks. Seventeen percentage of patients experienced ≥3 drug-related AEs, and there were no drug-related deaths, which indicated that Pembrolizumab was well-tolerated (14).

In the phase II trial KEYNOTE-055, 171 patients with progressed HNSCC within 6 months were treated with pembrolizumab. Among all patients, the HPV positive rate was 22%, and the PD-L1 positive rate was 82%. However, the ORR was 16%, regardless of HPV or PD-L1 status. The mOS was 8 months, and mPFS was 2.1 months. This trial may suggest that the therapeutic benefit of pembrolizumab is not limited to PD-L1 expression level or HPV status, but the validation of broad sample data is still needed (96).

In the phase III trial KEYNOTE-040, pembrolizumab or standard therapy was applied to 495 recurrent or metastatic HNSCC patients, whose disease progressed during or after platinum-based treatment. The ORR was 14.6 and 10.1% of pembrolizumab and standard therapy, respectively. The mOS was 8.4 and 6.9 months, while there was no difference in mPFS (2.1 vs. 2.3 months). Patients treated with pembrolizumab had fewer grade ≥3 AEs (13 vs. 36%). Most importantly, among patients with the PD-L1 protein tumor proportion score(TPS) ≥50%, pembrolizumab showed longer mOS (11.6 vs. 6.6 months), as well as mPFS (3.5 vs. 2.2 months) (15).

Following these studies, B. Burtness et al. conducted a phase III trial KEYNOTE-048 in which 882 patients were enrolling, comparing the curative effect of pembrolizumab or pembrolizumab combined with chemotherapy (P + C) vs. EXTREME (E, cetuximab + chemotherapy) for recurrent/metastatic HNSCC. Besides, this trial divided subgroups based on tumor PD-L1 expression levels (CPS ≥20 or CPS ≥1). Regardless of the PD-L1 degree, the E group had a better ORR (36 vs. 17%), but a higher incidence of grade ≥3 AEs (83 vs. 55%) than pembrolizumab monotherapy. Also, pembrolizumab monotherapy significantly improved OS over E in the PD-L1-positive populations but showed no difference in the total population. Moreover, this clinical trial suggested that PD-1 monotherapy could be applied for patients with PD-L1 positivity. However, for those patients with PD-L1-negative HNSCC, PD-1 antibody plus chemotherapy could achieve better survival benefits (16).

### Durvalumab

Durvalumab is one of the PD-L1 inhibitors which has not been approved for treating HNSCC by FDA, but the related clinical trials are underway (97). Segal et al. conducted a phase I/II trial that applied Durvalumab to 62 recurrent/metastatic HNSCC patients in a basket trial, including different solid tumor entities. The mOS was 8.4 months, and ORR was 12%. Drug-related AEs occurred in 59.7% and were grade 3–4 in 9.7%. However, mPFS did not differ by HPV status or smoking history in this trial, probably due to the insufficient sample size of patients (98).

Zandberg et al. conducted the phase II trial HAWK study, PD-L1-positive recurrent/metastatic HNSCC patients were treated with Durvalumab monotherapy. All enrolled patients (*n* = 112) had confirmed PD-L1-high expression (defined as ≥25% of tumor cells expressing PD-L1). ORR was 16.2% for all evaluable patients, 29.4% for HPV-positive patients and 10.9% for HPV-negative patients. The OS and mPFS were 7.1 and 2.1 months. Grade ≥3 AEs were 8.0%, and none led to death (18).

Conversely, the randomized, open-label, phase II CONDOR study, using Durvalumab, Tremelimumab (CTLA-4 inhibitors), and a combination of both to treat advanced HNSCC. PD-L1 tumor expression was low or negative in all 267 patients. ORR was 7.8, 9.2, and 1.6% in the combination arm, Durvalumab, and Tremelimumab; mOS for all patients treated was 7.6, 6.0, and 5.5 months, respectively. Grade ≥3 AEs occurred in 15.8, 12.3, and 16.9% (99). The results of the above studies show that PD-L1 and HPV-positive patients are more likely to benefit from PD-1/PD-L1 blockade.

### Atezolizumab

Atezolizumab is another PD-L1 monoclonal antibody that can exert antitumor effects through blocking PD-L1. In a phase I basket trial, Atezolizumab was applied to treat patients with advanced solid malignancies or hematologic malignancies, including thirty-two HNSCC patients whose disease was previously treated and then advanced. With no regard to HPV status or PD-L1 expression, the ORR was 22%. Besides, mOS was 6.0 months, and mPFS was 2.6 months. The incidence of grade ≥3 AEs was 13% (100).

### Combination Therapy of HPV Vaccine and PD-1 Blockade

Although patients with HPV-positive HNSCC have a relatively higher response rate to PD-1/PD-L1 inhibitors, it still does not exceed 30% (14, 18, 101). As mentioned before, most patients with HPV-positive tumors exhibited a more robust adaptive immune response triggered by HPV antigens (102). Therefore, some studies have explored whether combination with HPV vaccines could increase the response rate of PD-1/PD-L1 inhibitors in patients with HNSCC so that more people can benefit from this. Sun et al. found that PD-L1 blockade enhanced the anti-tumor immune response of an E7 antigen-specific protein vaccine *in vivo* by mediating M1-like polarization in macrophages and DCs development (103). A study found that E6/E7-targeted vaccine treatment can expand tumor-specific T cells and reduce tumor burden. Tan et al. indicated that combination therapy of the E6/E7-targeted vaccine and PD-L1 antagonist could more effectively control tumor growth and improve the survival of mice with HNSCC (104). Another study also showed that the combination of HPV E6/E7 vaccines and PD-1 inhibition could effectively inhibit tumor growth and reduce PD-L1 expression (105). In phase II clinical trial, 24 patients with HPV-positive cancer were treated with therapeutic HPV vaccines and PD-1 antagonist—Nivolumab. The ORR was 33%, and mOS was 17.5 months, which was better than PD-1 inhibitors alone in similar patients (106). Although the response rate had been improved slightly by this combination therapy, there were still a large number of patients who did not respond, suggesting the underlying mechanism is worthy of further exploration, and randomized clinical trials with more evaluable patients are also needed.

## Conclusion and Perspective

Recently, PD-1/PD-L1 inhibitors have been widely approved by the FDA for a variety of solid tumors and achieved inspiring outcomes. For patients with advanced HNSCC that are ineffective with traditional therapy, PD-1 or PD-L1 monoclonal antibodies show their advantages, with a moderate response and better drug-tolerance. However, there are still a majority of patients who could not benefit from these inhibitors. PD-L1 protein is over-expressed in HNSCC tissues above 50%, but always only 15% of patients can respond. Even some studies have shown disease progression after the treatment of the PD-1 antibody. In a study of recurrent or metastatic HNSCC, some patients treated with PD-1 inhibitors experience hyper-progression, which was associated with locoregional recurrence and shorter progression-free survival (107). For oral premalignant lesions, the animal model showed that although the PD-1 antibody stabilizes the disease at an early stage, after a while, it still failed with continued lesion progression (108). However, it is worth noting that the moderate ORR of PD-1/PD-L1 inhibitors may be the result of the application for the treatment of advanced HNSCC patients, where traditional treatment options have failed and are unlikely to improve the outcomes by further treatment.

Therefore, it is crucial to understand whether other inhibitory molecular pathways cooperate with PD-1/PD-L1 to maintain suppressed cellular immunity, and clarifying the cross-talk between these pathways and maximizing the effectiveness of PD-1 inhibitors are worthy of further study. For example, Tim-3 represents a highly dysfunctional state of TILs. A study found that PD-1/PD-L1 blockade resulted in further Tim-3 up-regulation on TILs through PI3K/Akt pathway, leading to immune escape and adaptive resistance to PD-1 or PD-L1 monotherapy (109). Similarly, TIGIT/CD155 pathway also contributes to the “exhaustion” state of TILs. TIGIT was over-expressed on Tregs of HNSCC patients and mouse models, and PD-1/PD-L1 blockade up-regulated TIGHT expression on Tregs, which was associated with immune suppression (110). It was reported that PD-1 blockade combined with TLR agonists could activate TAMs and induces tumor-specific adaptive immune responses (111). Moreover, PD-1/PD-L1 blockade enhances cetuximab-based cancer immunotherapy and reverses CD8^+^ TILs dysfunction (112). Down-regulation of major histocompatibility complex (MHC) class I is a tumor immune evasion mechanism. MEK inhibitor Trametinib could enhance both MHC class I and PD-L1 expression in human HNSCC cell lines, which was mediated by STAT3 activation. At the same time, combined Trametinib with PD-L1 blockade increased CD8^+^ T cell infiltration in the tumor microenvironment and delayed tumor growth (113).

Additionally, considering the cost and toxicity of immune therapy, it is necessary to search for stable and useful biomarkers, including the PD-1 and PD-L1 expression level, HPV status, tumor mutation burden (TMB) and immune infiltration situation, etc., in order to select the most appropriate individuals to administer the drug and optimize the therapeutic regimen (114–118). Meanwhile, more indications for the use of PD-1/PD-L1 inhibitors in HNC, such as early stages HNSCC or salivary gland tumors are needed for further investigation.

In summary, there is a growing interest in the mechanism of the PD-1/PD-L1 pathway involved in tumor progression, and immune checkpoint blockade has achieved initial success in patients with recurrent or/and metastatic HNSCC. Understanding the expression of PD-1 and PD-L1 in tumor cells is beneficial to our understanding of the biological behavior of HNSCC. Still, their expression and function in immune-infiltrating lymphocytes also need further study. Further research shouldwork toward finding populations that can respond to PD-1/PD-L1 blockade and combining with other molecular targets to improve the response rate and prolong response duration.

## Author Contributions

X-wQ and JJ were mainly responsible for the manuscript writing. XP, M-cH, and Y-jT were assisted in writing. X-hL and Y-lT provided suggestions on the ideas and performed the final corrections.

## Conflict of Interest

The authors declare that the research was conducted in the absence of any commercial or financial relationships that could be construed as a potential conflict of interest.

## References

[1] Dorsey K Agulnik M. Promising new molecular targeted therapies in head and neck cancer. Drugs. (2013) 73:315–25. 10.1007/s40265-013-0025-3 23440867

[2] Torre LA Bray F Siegel RL Ferlay J Lortet-Tieulent J Jemal A. Global cancer statistics, 2012. CA Cancer J Clin. (2015) 65:87–108. 10.3322/caac.21262 25651787

[3] Haddad RI Shin DM. Recent advances in head and neck cancer. N Engl J Med. (2008) 359:1143–54. 10.1056/NEJMra0707975 18784104

[4] Leemans CR Braakhuis BJ Brakenhoff RH The molecular biology of head and neck cancer. Nat Rev Cancer. (2011) 11:9–22. 10.1038/nrc2982 21160525

[5] Chi AC Day TA Neville BW. Oral cavity and oropharyngeal squamous cell carcinoma–an update. CA Cancer J Clin. (2015) 65:401–21. 10.3322/caac.21293 26215712

[6] Rothschild U Muller L Lechner A Schlösser HA Beutner D Läubli H. Immunotherapy in head and neck cancer–scientific rationale, current treatment options and future directions. Swiss Med Wkly. (2018) 148:w14625. 10.4414/smw.2018.14625 29756633

[7] Cramer JD Burtness B Ferris RL. Immunotherapy for head and neck cancer: recent advances and future directions. Oral Oncol. (2019) 99:104460. 10.1016/j.oraloncology.2019.104460 31683169 PMC7749717

[8] Ferris RL Immunology and immunotherapy of head and neck cancer. J Clin Oncol. (2015) 33:3293–304. 10.1200/JCO.2015.61.1509 26351330 PMC4586169

[9] Hodi FS Chiarion-Sileni V Gonzalez R Grob JJ Rutkowski P Cowey CL. Nivolumab plus ipilimumab or nivolumab alone versus ipilimumab alone in advanced melanoma (CheckMate 067): 4-year outcomes of a multicentre, randomised, phase 3 trial. Lancet Oncol. (2018) 19:1480–92. 10.1016/S1470-2045(18)30700-9 30361170

[10] Burtness B Haddad R Dinis J Trigo J Yokota T de Souza Viana L. Afatinib vs placebo as adjuvant therapy after chemoradiotherapy in squamous cell carcinoma of the head and neck: a randomized clinical trial. JAMA Oncol. (2019) 5:1170–80. 10.1001/jamaoncol.2019.1146 31194247 PMC6567846

[11] Tran L Allen CT Xiao R Moore E Davis R Park SJ. Cisplatin alters antitumor immunity and synergizes with PD-1/PD-L1 inhibition in head and neck squamous cell carcinoma. Cancer Immunol Res. (2017) 5:1141–51. 10.1158/2326-6066.CIR-17-0235 29097421 PMC5712281

[12] Addeo R Ghiani M Merlino F Ricciardiello F Caraglia M. CheckMate 141 trial: all that glitters is not gold. Expert Opin Biol Ther. (2019) 19:169–71. 10.1080/14712598.2019.1570498 30652499

[13] Ferris RL Blumenschein G Jr Fayette J Guigay J Colevas AD Licitra L. Nivolumab for recurrent squamous-cell carcinoma of the head and neck. N Engl J Med. (2016) 375:1856–67. 10.1056/NEJMoa1602252 27718784 PMC5564292

[14] Seiwert TY Burtness B Mehra R Weiss J Berger R Eder JP. Safety and clinical activity of pembrolizumab for treatment of recurrent or metastatic squamous cell carcinoma of the head and neck (KEYNOTE-012): an open-label, multicentre, phase 1b trial. Lancet Oncol. (2016) 17:956–65. 10.1016/S1470-2045(16)30066-3 27247226

[15] Cohen EEW Soulières D Le Tourneau C Dinis J Licitra L Ahn MJ Pembrolizumab versus methotrexate, docetaxel, or cetuximab for recurrent or metastatic head-and-neck squamous cell carcinoma (KEYNOTE-040): a randomised, open-label, phase 3 study. Lancet. (2019) 393:156–67. 10.1016/S0140-6736(18)31999-8 30509740

[16] Burtness B Harrington KJ Greil R Soulières D Tahara M de Castro G Jr Pembrolizumab alone or with chemotherapy versus cetuximab with chemotherapy for recurrent or metastatic squamous cell carcinoma of the head and neck (KEYNOTE-048): a randomised, open-label, phase 3 study. Lancet. (2019) 394:1915–28. 10.1016/S0140-6736(19)32591-7 31679945

[17] Ribas A Wolchok JD. Cancer immunotherapy using checkpoint blockade. Science. (2018) 359:1350–5. 10.1126/science.aar4060 29567705 PMC7391259

[18] Zandberg DP Algazi AP Jimeno A Good JS Fayette J Bouganim N. Durvalumab for recurrent or metastatic head and neck squamous cell carcinoma: Results from a single-arm, phase II study in patients with ≥25% tumour cell PD-L1 expression who have progressed on platinum-based chemotherapy. Eur J Cancer. (2019) 107:142–52. 10.1016/j.ejca.2018.11.015 30576970

[19] Okazaki T Honjo T. PD-1 and PD-1 ligands: from discovery to clinical application. Int Immunol. (2007) 19:813–24. 10.1093/intimm/dxm057 17606980

[20] Keir ME Butte MJ Freeman GJ Sharpe AH. PD-1 and its ligands in tolerance and immunity. Annu Rev Immunol. (2008) 26:677–704. 10.1146/annurev.immunol.26.021607.090331 18173375 PMC10637733

[21] Dong H Zhu G Tamada K Chen L. B7-H1, a third member of the B7 family, co-stimulates T-cell proliferation and interleukin-10 secretion. Nat Med. (1999) 5:1365–9. 10.1038/70932 10581077

[22] Buderath P Schwich E Jensen C Horn PA Kimmig R Kasimir-Bauer S. Soluble programmed death receptor ligands sPD-L1 and sPD-L2 as liquid biopsy markers for prognosis and platinum response in epithelial ovarian cancer. Front Oncol. (2019) 9:1015. 10.3389/fonc.2019.01015 31681568 PMC6803523

[23] Cubillos-Zapata C Martínez-García M Campos-Rodríguez F Sánchez de la Torre M Nagore E. Soluble PD-L1 is a potential biomarker of cutaneous melanoma aggressiveness and metastasis in obstructive sleep apnoea patients. Eur Respir J. (2019) 53:1801298. 10.1183/13993003.01298-2018 30487198

[24] Wang Q Zhang J Tu H Liang D Chang DW Ye Y. Soluble immune checkpoint-related proteins as predictors of tumor recurrence, survival, and T cell phenotypes in clear cell renal cell carcinoma patients. J Immunother Cancer. (2019) 7:334. 10.1186/s40425-019-0810-y 31783776 PMC6884764

[25] Zhang LQ Chen Y Pan X Xing YF Shi MH Chen YJ. Level of soluble programmed death-1 ligand 1 in peripheral blood of patients with advanced epidermal growth factor receptor mutated lung adenocarcinoma and its clinical implications. Zhonghua Yi Xue Za Zhi. (2016) 96:3870–74. 10.3760/cma.j.issn.0376-2491.2016.48.004 28057155

[26] Theodoraki MN Yerneni SS Hoffmann TK Gooding WE Whiteside TL. Clinical significance of PD-L1(+) exosomes in plasma of head and neck cancer patients. Clin Cancer Res. (2018) 24:896–905. 10.1158/1078-0432.CCR-17-2664 29233903 PMC6126905

[27] Butte MJ Keir ME Phamduy TB Sharpe AH Freeman GJ. Programmed death-1 ligand 1 interacts specifically with the B7-1 costimulatory molecule to inhibit T cell responses. Immunity. (2007) 27:111–22. 10.1016/j.immuni.2007.05.016 17629517 PMC2707944

[28] Park JJ Omiya R Matsumura Y Sakoda Y Kuramasu A Augustine MM. B7-H1/CD80 interaction is required for the induction and maintenance of peripheral T-cell tolerance. Blood. (2010) 116:1291–8. 10.1182/blood-2010-01-265975 20472828 PMC2938239

[29] Yearley JH Gibson C Yu N Moon C Murphy E Juco J. PD-L2 expression in human tumors: relevance to anti-PD-1 therapy in cancer. Clin Cancer Res. (2017) 23:3158–67. 10.1158/1078-0432.CCR-16-1761 28619999

[30] Messal N Serriari NE Pastor S Nunès JA Olive D. PD-L2 is expressed on activated human T cells and regulates their function. Mol Immunol. (2011) 48:2214–9. 10.1016/j.molimm.2011.06.436 21752471

[31] Latchman Y Wood CR Chernova T Chaudhary D Borde M Chernova I. PD-L2 is a second ligand for PD-1 and inhibits T cell activation. Nat Immunol. (2001) 2:261–8. 10.1038/85330 11224527

[32] Rodig N Ryan T Allen JA Pang H Grabie N Chernova T. Endothelial expression of PD-L1 and PD-L2 down-regulates CD8^+^ T cell activation and cytolysis. Eur J Immunol. (2003) 33:3117–26. 10.1002/eji.200324270 14579280

[33] Schoenfeld JD Gjini E Rodig SJ Tishler RB Rawal B Catalano PJ. Evaluating the PD-1 axis and immune effector cell infiltration in oropharyngeal squamous cell carcinoma. Int J Radiat Oncol Biol Phys. (2018) 102:137–45. 10.1016/j.ijrobp.2018.05.002 29960819

[34] Ritprajak P. Azuma M Intrinsic and extrinsic control of expression of the immunoregulatory molecule PD-L1 in epithelial cells and squamous cell carcinoma. Oral Oncol. (2015) 51:221–8. 10.1016/j.oraloncology.2014.11.014 25500094

[35] Tsushima F Tanaka K Otsuki N Youngnak P Iwai H Omura K. Predominant expression of B7-H1 and its immunoregulatory roles in oral squamous cell carcinoma. Oral Oncol. (2006) 42:268–74. 10.1016/j.oraloncology.2005.07.013 16271509

[36] Chen J Feng Y Lu L Wang H Dai L Li Y. Interferon-γ-induced PD-L1 surface expression on human oral squamous carcinoma via PKD2 signal pathway. Immunobiology. (2012) 217:385–93. 10.1016/j.imbio.2011.10.016 22204817

[37] Xu C Fillmore CM Koyama S Wu H Zhao Y Chen Z. Loss of Lkb1 and Pten leads to lung squamous cell carcinoma with elevated PD-L1 expression. Cancer Cell. (2014) 25:590–604. 10.1016/j.ccr.2014.03.033 24794706 PMC4112370

[38] Chen L Yang QC Li YC Yang LL Liu JF Li H. Targeting CMTM6 suppresses stem cell-like properties and enhances antitumor immunity in head and neck squamous cell carcinoma. Cancer Immunol Res. (2020) 8:179–91. 10.1158/2326-6066.CIR-19-0394 31771985

[39] Burr ML Sparbier CE Chan YC Williamson JC Woods K Beavis PA. CMTM6 maintains the expression of PD-L1 and regulates anti-tumour immunity. Nature. (2017) 549:101–5. 10.1038/nature23643 28813417 PMC5706633

[40] Mezzadra R Sun C Jae LT Gomez-Eerland R de Vries E Wu W. Identification of CMTM6 and CMTM4 as PD-L1 protein regulators. Nature. (2017) 549:106–10. 10.1038/nature23669 28813410 PMC6333292

[41] Yu GT Bu LL Huang CF Zhang WF Chen WJ Gutkind JS. PD-1 blockade attenuates immunosuppressive myeloid cells due to inhibition of CD47/SIRPα axis in HPV negative head and neck squamous cell carcinoma. Oncotarget. (2015) 6:42067–80. 10.18632/oncotarget.5955 26573233 PMC4747210

[42] Strome SE Dong H Tamura H Voss SG Flies DB Tamada K. B7-H1 blockade augments adoptive T-cell immunotherapy for squamous cell carcinoma. Cancer Res. (2003) 63:6501–5. 14559843

[43] Mattox AK Lee J Westra WH Pierce RH Ghossein R Faquin WC. PD-1 expression in head and neck squamous cell carcinomas derives primarily from functionally anergic CD4(+) TILs in the presence of PD-L1(+) TAMs. Cancer Res. (2017) 77:6365–74. 10.1158/0008-5472.CAN-16-3453 28947422 PMC5690870

[44] Lyford-Pike S Peng S Young GD Taube JM Westra WH Akpeng B. Evidence for a role of the PD-1:PD-L1 pathway in immune resistance of HPV-associated head and neck squamous cell carcinoma. Cancer Res. (2013) 73:1733–41. 10.1158/0008-5472.CAN-12-2384 23288508 PMC3602406

[45] Rasmussen JH Lelkaitis G Håkansson K Vogelius IR Johannesen HH Fischer BM. Intratumor heterogeneity of PD-L1 expression in head and neck squamous cell carcinoma. Br J Cancer. (2019) 120:1003–6. 10.1038/s41416-019-0449-y 30967647 PMC6734649

[46] Kim HS Lee JY Lim SH Park K Sun JM Ko YH. Association between PD-L1 and HPV status and the prognostic value of PD-L1 in oropharyngeal squamous cell carcinoma. Cancer Res Treat. (2016) 48:527–36. 10.4143/crt.2015.249 26511814 PMC4843713

[47] Ou D Adam J Garberis I Blanchard P Nguyen F Levy A Clinical relevance of tumor infiltrating lymphocytes, PD-L1 expression and correlation with HPV/p16 in head and neck cancer treated with bio- or chemo-radiotherapy. Oncoimmunology. (2017) 6:e1341030 10.1080/2162402X.2017.1341030 28932643 PMC5599076

[48] Balermpas P Rödel F Krause M Linge A Lohaus F Baumann M. The PD-1/PD-L1 axis and human papilloma virus in patients with head and neck cancer after adjuvant chemoradiotherapy: a multicentre study of the German cancer consortium radiation oncology group (DKTK-ROG). Int J Cancer. (2017) 141:594–603. 10.1002/ijc.30770 28480996

[49] Hong AM Vilain RE Romanes S Yang J Smith E Jones D. PD-L1 expression in tonsillar cancer is associated with human papillomavirus positivity and improved survival: implications for anti-PD1 clinical trials. Oncotarget. (2016) 7:77010–20. 10.18632/oncotarget.12776 27776338 PMC5363566

[50] Hong AM Ferguson P Dodds T Jones D Li M Yang J. Significant association of PD-L1 expression with human papillomavirus positivity and its prognostic impact in oropharyngeal cancer. Oral Oncol. (2019) 92:33–9. 10.1016/j.oraloncology.2019.03.012 31010620

[51] Yang WF Wong MCM Thomson PJ Li KY Su YX. The prognostic role of PD-L1 expression for survival in head and neck squamous cell carcinoma: a systematic review and meta-analysis. Oral Oncol. (2018) 86:81–90. 10.1016/j.oraloncology.2018.09.016 30409325

[52] Lin YM Sung WW Hsieh MJ Tsai SC Lai HW Yang SM. High PD-L1 expression correlates with metastasis and poor prognosis in oral squamous cell carcinoma. PLoS ONE. (2015) 10:e0142656. 10.1371/journal.pone.0142656 26562534 PMC4642967

[53] Moratin J Metzger K Safaltin A Herpel E Hoffmann J Freier K. Upregulation of PD-L1 and PD-L2 in neck node metastases of head and neck squamous cell carcinoma. Head Neck. (2019) 41:2484–91. 10.1002/hed.25713 30821864

[54] Vassilakopoulou M Avgeris M Velcheti V Kotoula V Rampias T Chatzopoulos K Evaluation of PD-L1 cinoma. Clin Cancer Res. (2016) 22:704–13. 10.1158/1078-0432.CCR-15-1543 26408403

[55] Lyu X Zhang M Li G Jiang Y Qiao Q. PD-1 and PD-L1 expression predicts radiosensitivity and clinical outcomes in head and neck cancer and is associated with HPV infection. J Cancer. (2019) 10:937–48. 10.7150/jca.27199 30854100 PMC6400795

[56] Outh-Gauer S Alt M Le Tourneau C Augustin J Broudin C Gasne C. Immunotherapy in head and neck cancers: a new challenge for immunologists, pathologists and clinicians. Cancer Treat Rev. (2018) 65:54–64. 10.1016/j.ctrv.2018.02.008 29547766

[57] Kim HR Ha SJ Hong MH Heo SJ Koh YW Choi EC PD-L1 expression on immune cells, but not on tumor cells, is a favorable prognostic factor for head and neck cancer patients. Sci Rep. (2016) 6:36956 10.1038/srep36956 27841362 PMC5107906

[58] Hsu MC Hsiao JR Chang KC Wu YH Su IJ Jin YT. Increase of programmed death-1-expressing intratumoral CD8 T cells predicts a poor prognosis for nasopharyngeal carcinoma. Mod Pathol. (2010) 23:1393–403. 10.1038/modpathol.2010.130 20657553

[59] Jie HB Gildener-Leapman N Li J Srivastava RM Gibson SP Whiteside TL. Intratumoral regulatory T cells upregulate immunosuppressive molecules in head and neck cancer patients. Br J Cancer. (2013) 109:2629–35. 10.1038/bjc.2013.645 24169351 PMC3833228

[60] Badoual C Hans S Merillon N Van Ryswick C Ravel P Benhamouda N. PD-1-expressing tumor-infiltrating T cells are a favorable prognostic biomarker in HPV-associated head and neck cancer. Cancer Res. (2013) 73:128–38. 10.1158/0008-5472.CAN-12-2606 23135914

[61] Partlová S Bouček J Kloudová K Lukešová E Zábrodský M Grega M. Distinct patterns of intratumoral immune cell infiltrates in patients with HPV-associated compared to non-virally induced head and neck squamous cell carcinoma. Oncoimmunology. (2015) 4:e965570. 10.4161/21624011.2014.965570 25949860 PMC4368144

[62] Poropatich K Fontanarosa J Swaminathan S Dittmann D Chen S Samant S. Comprehensive T-cell immunophenotyping and next-generation sequencing of human papillomavirus (HPV)-positive and HPV-negative head and neck squamous cell carcinomas. J Pathol. (2017) 243:354–65. 10.1002/path.4953 28771750

[63] Carrero I Liu HC Sikora AG Milosavljevic A. Histoepigenetic analysis of HPV- and tobacco-associated head and neck cancer identifies both subtype-specific and common therapeutic targets despite divergent microenvironments. Oncogene. (2019) 38:3551–68. 10.1038/s41388-018-0659-4 30655605 PMC6756123

[64] Kansy BA Concha-Benavente F Srivastava RM Jie HB Shayan G Lei Y. PD-1 status in CD8(+) T cells associates with survival and anti-PD-1 therapeutic outcomes in head and neck cancer. Cancer Res. (2017) 77:6353–64. 10.1158/0008-5472.CAN-16-3167 28904066 PMC5690836

[65] Czystowska M Gooding W Szczepanski MJ Lopez-Abaitero A Ferris RL Johnson JT. The immune signature of CD8(+)CCR7(+) T cells in the peripheral circulation associates with disease recurrence in patients with HNSCC. Clin Cancer Res. (2013) 19:889–99. 10.1158/1078-0432.CCR-12-2191 23363813 PMC3708459

[66] Li J Shayan G Avery L Jie HB Gildener-Leapman N Schmitt N. Tumor-infiltrating Tim-3(+) T cells proliferate avidly except when PD-1 is co-expressed: Evidence for intracellular cross talk. Oncoimmunology. (2016) 5:e1200778. 10.1080/2162402X.2016.1200778 27853635 PMC5087305

[67] Blackburn SD Shin H Haining WN Zou T Workman CJ Polley A. Coregulation of CD8^+^ T cell exhaustion by multiple inhibitory receptors during chronic viral infection. Nat Immunol. (2009) 10:29–37. 10.1038/ni.1679 19043418 PMC2605166

[68] Malm IJ Bruno TC Fu J Zeng Q Taube JM Westra W. Expression profile and *in vitro* blockade of programmed death-1 in human papillomavirus-negative head and neck squamous cell carcinoma. Head Neck. (2015) 37:1088–95. 10.1002/hed.23706 24710745 PMC4390546

[69] Lechner A Schlößer H Rothschild SI Thelen M Reuter S Zentis P. Characterization of tumor-associated T-lymphocyte subsets and immune checkpoint molecules in head and neck squamous cell carcinoma. Oncotarget. (2017) 8:44418–33. 10.18632/oncotarget.17901 28574843 PMC5546490

[70] Montler R Bell RB Thalhofer C Leidner R Feng Z Fox BA. OX40 PD-1 and CTLA-4 are selectively expressed on tumor-infiltrating T cells in head and neck cancer. Clin Transl Immunol. (2016) 5:e70. 10.1038/cti.2016.16 27195113 PMC4855266

[71] Parry RV Chemnitz JM Frauwirth KA Lanfranco AR Braunstein I Kobayashi SV. CTLA-4 and PD-1 receptors inhibit T-cell activation by distinct mechanisms. Mol Cell Biol. (2005) 25:9543–53. 10.1128/MCB.25.21.9543-9553.2005 16227604 PMC1265804

[72] Chen L. Co-inhibitory molecules of the B7-CD28 family in the control of T-cell immunity. Nat Rev Immunol. (2004) 4:336–47. 10.1038/nri1349 15122199

[73] Francisco LM Sage PT Sharpe AH. The PD-1 pathway in tolerance and autoimmunity. Immunol Rev. (2010) 236:219–42. 10.1111/j.1600-065X.2010.00923.x 20636820 PMC2919275

[74] Francisco LM Salinas VH Brown KE Vanguri VK Freeman GJ Kuchroo VK. PD-L1 regulates the development, maintenance, and function of induced regulatory T cells. J Exp Med. (2009) 206:3015–29. 10.1084/jem.20090847 20008522 PMC2806460

[75] Liu Z McMichael EL Shayan G Li J Chen K Srivastava R. Novel effector phenotype of Tim-3(+) regulatory t cells leads to enhanced suppressive function in head and neck cancer patients. Clin Cancer Res. (2018) 24:4529–38. 10.1158/1078-0432.CCR-17-1350 29712685 PMC6139056

[76] Concha-Benavente F Kansy B Moskovitz J Moy J Chandran U Ferris RL. PD-L1 mediates dysfunction in activated PD-1(+) NK cells in head and neck cancer patients. Cancer Immunol Res. (2018) 6:1548–60. 10.1158/2326-6066.CIR-18-0062 30282672 PMC6512340

[77] Makowska A Braunschweig T Denecke B Shen L Baloche V Busson P. Interferon β and anti-PD-1/PD-L1 checkpoint blockade cooperate in NK cell-mediated killing of nasopharyngeal carcinoma cells. Transl Oncol. (2019) 12:1237–56. 10.1016/j.tranon.2019.04.017 31295651 PMC6617170

[78] Mantovani A Sica A Macrophages innate immunity and cancer: balance tolerance and diversity Curr Opin Immunol. (2010) 22:231–7. 10.1016/j.coi.2010.01.009 20144856

[79] Hartley GP Chow L Ammons DT Wheat WH Dow SW. Programmed cell death ligand 1 (PD-L1) signaling regulates macrophage proliferation and activation. Cancer Immunol Res. (2018) 6:1260–73. 10.1158/2326-6066.CIR-17-0537 30012633

[80] Hirai M Kitahara H Kobayashi Y Kato K Bou-Gharios G Nakamura H. Regulation of PD-L1 expression in a high-grade invasive human oral squamous cell carcinoma microenvironment. Int J Oncol. (2017) 50:41–8. 10.3892/ijo.2016.3785 27922697 PMC5182007

[81] Jiang C Yuan F Wang J Wu L. Oral squamous cell carcinoma suppressed antitumor immunity through induction of PD-L1 expression on tumor-associated macrophages. Immunobiology. (2017) 222:651–7. 10.1016/j.imbio.2016.12.002 28017495

[82] Kubota K Moriyama M Furukawa S Rafiul H Maruse Y Jinno T. CD163(+)CD204(+) tumor-associated macrophages contribute to T cell regulation via interleukin-10 and PD-L1 production in oral squamous cell carcinoma. Sci Rep. (2017) 7:1755. 10.1038/s41598-017-01661-z 28496107 PMC5431876

[83] Baruah P Bullenkamp J Wilson POG Lee M Kaski JC Dumitriu IE. TLR9 mediated tumor-stroma interactions in human papilloma virus (HPV)-positive head and neck squamous cell carcinoma up-regulate PD-L1 and PD-L2. Front Immunol. (2019) 10:1644. 10.3389/fimmu.2019.01644 31379843 PMC6648892

[84] Cho YA Yoon HJ Lee JI Hong SP Hong SD. Relationship between the expressions of PD-L1 and tumor-infiltrating lymphocytes in oral squamous cell carcinoma. Oral Oncol. (2011) 47:1148–53. 10.1016/j.oraloncology.2011.08.007 21911310

[85] Nazareth MR Broderick L Simpson-Abelson MR Kelleher RJ Jr Yokota SJ Bankert RB. Characterization of human lung tumor-associated fibroblasts and their ability to modulate the activation of tumor-associated T cells. J Immunol. (2007) 178:5552–62. 10.4049/jimmunol.178.9.5552 17442937

[86] Kang JH Jung MY Choudhury M Leof EB. Transforming growth factor beta induces fibroblasts to express and release the immunomodulatory protein PD-L1 into extracellular vesicles. Faseb J. (2020) 34:2213–26. 10.1096/fj.201902354R 31907984

[87] Dong H Strome SE Salomao DR Tamura H Hirano F Flies DB. Tumor-associated B7-H1 promotes T-cell apoptosis: a potential mechanism of immune evasion. Nat Med. (2002) 8:793–800. 10.1038/nm730 12091876

[88] Prince ME Sivanandan R Kaczorowski A Wolf GT Kaplan MJ Dalerba P. Identification of a subpopulation of cells with cancer stem cell properties in head and neck squamous cell carcinoma. Proc Natl Acad Sci USA. (2007) 104:973–8. 10.1073/pnas.0610117104 17210912 PMC1783424

[89] Lee Y Shin JH Longmire M Wang H Kohrt HE Chang HY. CD44^+^ cells in head and neck squamous cell carcinoma suppress T-cell-mediated immunity by selective constitutive and inducible expression of PD-L1. Clin Cancer Res. (2016) 22:3571–81. 10.1158/1078-0432.CCR-15-2665 26864211 PMC5623594

[90] Guay C Regazzi R. Exosomes as new players in metabolic organ cross-talk. Diabetes Obes Metab. (2017) 19:137–46. 10.1111/dom.13027 28880477

[91] Ludwig S Floros T Theodoraki MN Hong CS Jackson EK Lang S. Suppression of lymphocyte functions by plasma exosomes correlates with disease activity in patients with head and neck cancer. Clin Cancer Res. (2017) 23:4843–54. 10.1158/1078-0432.CCR-16-2819 28400428 PMC5559308

[92] Whiteside TL. Exosomes and tumor-mediated immune suppression. J Clin Invest. (2016) 126:1216–23. 10.1172/JCI81136 26927673 PMC4811135

[93] Theodoraki MN Hoffmann TK Jackson EK Whiteside TL. Exosomes in HNSCC plasma as surrogate markers of tumour progression and immune competence. Clin Exp Immunol. (2018) 194:67–78. 10.1111/cei.13157 30229863 PMC6156813

[94] Theodoraki MN Hoffmann TK Whiteside TL. Separation of plasma-derived exosomes into CD3((+)) and CD3((-)) fractions allows for association of immune cell and tumour cell markers with disease activity in HNSCC patients. Clin Exp Immunol. (2018) 192:271–83. 10.1111/cei.13113 29431869 PMC5980445

[95] Ferris RL Blumenschein G Jr Fayette J Guigay J Colevas AD. Nivolumab vs investigator's choice in recurrent or metastatic squamous cell carcinoma of the head and neck: 2-year long-term survival update of checkMate 141 with analyses by tumor PD-L1 expression. Oral Oncol. (2018) 81:45–51. 10.1016/j.oraloncology.2018.04.008 29884413 PMC6563923

[96] Bauml J Seiwert TY Pfister DG Worden F Liu SV Gilbert J. Pembrolizumab for platinum- and cetuximab-refractory head and neck cancer: results from a single-arm, phase II study. J Clin Oncol. (2017) 35:1542–9. 10.1200/JCO.2016.70.1524 28328302 PMC5946724

[97] Ibrahim R Stewart R Shalabi A. PD-L1 blockade for cancer treatment: MEDI4736. Semin Oncol. (2015) 42:474–83. 10.1053/j.seminoncol.2015.02.007 25965366

[98] Segal NH Ou SI Balmanoukian A Fury MG Massarelli E Brahmer JR. Safety and efficacy of durvalumab in patients with head and neck squamous cell carcinoma: results from a phase I/II expansion cohort. Eur J Cancer. (2019) 109:154–61. 10.1016/j.ejca.2018.12.029 30731276

[99] Siu LL Even C Mesía R Remenar E Daste A Delord JP. Safety and efficacy of durvalumab with or without tremelimumab in patients with PD-L1-Low/Negative recurrent or metastatic HNSCC: the phase 2 CONDOR randomized clinical trial. JAMA Oncol. (2019) 5:195–203. 10.1001/jamaoncol.2018.4628 30383184 PMC6439564

[100] Colevas AD Bahleda R Braiteh F Balmanoukian A Brana I Chau NG. Safety and clinical activity of atezolizumab in head and neck cancer: results from a phase I trial. Ann Oncol. (2018) 29:2247–53. 10.1093/annonc/mdy411 30219915

[101] Hanna GJ Lizotte P Cavanaugh M Kuo FC Shivdasani P Frieden A. Frameshift events predict anti-PD-1/L1 response in head and neck cancer. JCI Insight. (2018) 3:e98811. 10.1172/jci.insight.98811 29467336 PMC5916245

[102] Welters MJP Ma W Santegoets S Goedemans R Ehsan I Jordanova ES. Intratumoral HPV16-specific T cells constitute a type i-oriented tumor microenvironment to improve survival in HPV16-driven oropharyngeal cancer. Clin Cancer Res. (2018) 24:634–47. 10.1158/1078-0432.CCR-17-2140 29018052

[103] Sun NY Chen YL Wu WY Lin HW Chiang YC Chang CF. Blockade of PD-L1 enhances cancer immunotherapy by regulating dendritic cell maturation and macrophage polarization. Cancers (Basel). (2019) 11:1400. 10.3390/cancers11091400 31546897 PMC6769724

[104] Tan YS Sansanaphongpricha K Xie Y Donnelly CR Luo X Heath BR. Mitigating SOX2-potentiated immune escape of head and neck squamous cell carcinoma with a STING-inducing nanosatellite vaccine. Clin Cancer Res. (2018) 24:4242–55. 10.1158/1078-0432.CCR-17-2807 29769207 PMC6125216

[105] Rice AE Latchman YE Balint JP Lee JH Gabitzsch ES Jones FR. An HPV-E6/E7 immunotherapy plus PD-1 checkpoint inhibition results in tumor regression and reduction in PD-L1 expression. Cancer Gene Ther. (2015) 22:454–62. 10.1038/cgt.2015.40 26337747

[106] Massarelli E William W Johnson F Kies M Ferrarotto R Guo M. Combining immune checkpoint blockade and tumor-specific vaccine for patients with incurable human papillomavirus 16-related cancer: a phase 2 clinical trial. JAMA Oncol. (2019) 5:67–73. 10.1001/jamaoncol.2018.4051 30267032 PMC6439768

[107] Saâda-Bouzid E Defaucheux C Karabajakian A Coloma VP Servois V Paoletti X Hyperprogression during anti-PD-1/PD-L1 therapy in patients with recurrent and/or metastatic head and neck squamous cell carcinoma. Ann Oncol. (2017) 28:1605–11. 10.1093/annonc/mdx178 28419181

[108] Levingston CA Young MRI. Local immune responsiveness of mice bearing premalignant oral lesions to PD-1 antibody treatment. Cancers (Basel). (2017) 9:62. 10.3390/cancers9060062 28574425 PMC5483881

[109] Shayan G Srivastava R Li J Schmitt N Kane LP Ferris RL. Adaptive resistance to anti-PD1 therapy by Tim-3 upregulation is mediated by the PI3K-Akt pathway in head and neck cancer. Oncoimmunology. (2017) 6:e1261779. 10.1080/2162402X.2016.1261779 28197389 PMC5283618

[110] Wu L Mao L Liu JF Chen L Yu GT Yang LL. Blockade of TIGIT/CD155 signaling reverses T-cell exhaustion and enhances antitumor capability in head and neck squamous cell carcinoma. Cancer Immunol Res. (2019) 7:1700–13. 10.1158/2326-6066.CIR-18-0725 31387897

[111] Sato-Kaneko F Yao S Ahmadi A Zhang SS Hosoya T Kaneda MM. Combination immunotherapy with TLR agonists and checkpoint inhibitors suppresses head and neck cancer. JCI Insight. (2017) 2:e93397. 10.1172/jci.insight.93397 28931759 PMC5621908

[112] Jie HB Srivastava RM Argiris A Bauman JE Kane LP Ferris RL. Increased PD-1(+) and TIM-3(+) TILs during cetuximab therapy inversely correlate with response in head and neck cancer patients. Cancer Immunol Res. (2017) 5:408–16. 10.1158/2326-6066.CIR-16-0333 28408386 PMC5497750

[113] Kang SH Keam B Ahn YO Park HR Kim M Kim TM. Inhibition of MEK with trametinib enhances the efficacy of anti-PD-L1 inhibitor by regulating anti-tumor immunity in head and neck squamous cell carcinoma. Oncoimmunology. (2019) 8:e1515057. 10.1080/2162402X.2018.1515057 30546955 PMC6287796

[114] Oliva M Spreafico A Taberna M Alemany L Coburn B Mesia R. Immune biomarkers of response to immune-checkpoint inhibitors in head and neck squamous cell carcinoma. Ann Oncol. (2019) 30:57–67. 10.1093/annonc/mdy507 30462163 PMC6336003

[115] Schalper KA Kaftan E Herbst RS. Predictive biomarkers for PD-1 axis therapies: the hidden treasure or a call for research. Clin Cancer Res. (2016) 22:2102–4. 10.1158/1078-0432.CCR-16-0169 26957559 PMC4940186

[116] Solomon B Young RJ Rischin D. Head and neck squamous cell carcinoma: genomics and emerging biomarkers for immunomodulatory cancer treatments. Semin Cancer Biol. (2018) 52:228–40. 10.1016/j.semcancer.2018.01.008 29355614

[117] Wang J Sun H Zeng Q Guo XJ Wang H Liu HH. HPV-positive status associated with inflamed immune microenvironment and improved response to anti-PD-1 therapy in head and neck squamous cell carcinoma. Sci Rep. (2019) 9:13404. 10.1038/s41598-019-49771-0 31527697 PMC6746709

[118] Havel JJ Chowell D Chan TA. The evolving landscape of biomarkers for checkpoint inhibitor immunotherapy. Nat Rev Cancer. (2019) 19:133–50. 10.1038/s41568-019-0116-x 30755690 PMC6705396

